# Unraveling the “golden ratio”: a pilot study investigating acute-to-chronic workload ratio in breast cancer patients undergoing active treatment

**DOI:** 10.3389/fphys.2023.1273624

**Published:** 2024-01-08

**Authors:** Apostolos Z. Skouras, Dimitrios Antonakis-Karamintzas, Charilaos Tsolakis, Panagiotis Koulouvaris

**Affiliations:** ^1^ Sports Excellence, 1st Department of Orthopaedic Surgery, School of Medicine, National and Kapodistrian University of Athens, Athens, Greece; ^2^ Sports Performance Laboratory, School of Physical Education & Sports Science, National and Kapodistrian University of Athens, Athens, Greece

**Keywords:** breast cancer, exercise oncology, training load monitoring, ACWR ratio, undergoing cancer treatment

## Abstract

Training load monitoring is a common practice in sports medicine for supporting athletes’ health and performance. Despite progress in exercise oncology research for breast cancer patients, training load monitoring is underutilized. This study retrospectively investigated the relationship between maintained training load within a defined range and physical and health outcomes of ten breast cancer patients during active anticancer treatment who underwent a 12-week exercise program. Intervention consisted of endurance and resistance training, three times a week, with each session lasting 30–45 min. Assessments were conducted at baseline, 6 and 12 weeks after enrollment, evaluating physical function (6-min walk test–6MWT, and sit-to-stand), muscle strength, body composition, sleep quality (Pittsburgh Sleep Quality Index–Pittsburgh Sleep Quality Index), quality of life (EORTC-QLQ-C30), heart rate variability and physical activity levels (International Physical Activity Questionnaire–International Physical Activity Questionnaire). The Physiological Cost Index/Energy Expenditure Index (PCI/EEI) was estimated using the 6MWT and Heart rate. Training load monitoring was performed by session rating of perceived exertion (sRPE, relative intensity multiplying with session duration). Acute-to-Chronic Workload Ratio (ACWR) (7:28, rolling average) was calculated accordingly. Analyses were performed within-subjects across time points and between-subjects, comparing those who maintained from weeks 6–12 an ACWR of 0.8–1.3 with those who did not. Adherence rates were similar between groups. Physical function improved in the total sample with large effect sizes (Δ6MWT = 56.5 m [95%CI: 6–100 m], effect size [w] = 0.52, *p* = 0.006; ΔSit-to-Stand = 1.5 [95%CI: 1–5], effect size [w] = 0.681, *p* < 0.001), demonstrating greater changes in patients with higher ACWR. Sleep quality improvements were higher in the appropriate ACWR group (*p* = 0.016). A positive correlation was demonstrated between global health status and 6MWT change from baseline to 12 weeks (*ρ* = 0.689, *p* = 0.04). Despite a small sample size, patients maintaining sufficient relative training load presented greater physical fitness and sleep quality improvements. Thus, training load monitoring may enhance exercise program benefits in breast cancer patients under active treatment.

## 1 Introduction

Breast cancer ranks as the leading cancer type with the highest number of new cases annually worldwide (Arnold et al., 2022; Siegel et al., 2023). While early detection and targeted anticancer therapies have increased survivorship rates, therapy-induced adverse events are still burdensome and intractable in clinical oncology. These adverse events significantly affect the patient’s quality of life (QoL). Among the most common anticancer therapy-induced signs and symptoms are cardiotoxicity, reduced physical fitness, increased cancer-related fatigue (CRF), and sleep disturbances (Ansari et al., 2017; Palesh et al., 2018; Mallard et al., 2023). Several non-pharmacological treatments, like lifestyle modifications, have addressed such adverse events. To date, exercise is one of the most well-researched conservative modalities for mitigating breast cancer patients’ adverse events (Loprinzi and Cardinal, 2012; Palesh et al., 2018; Gaytan et al., 2023). Indeed, exercise interventions may enhance physical functioning, improve body composition, QoL and autonomic dysfunction, and reduce CRF during chemotherapy or radiotherapy in breast cancer patients (Courneya et al., 2007; Furmaniak et al., 2016; Lavín-Pérez et al., 2021). Different aerobic and resistance training combinations have been applied in breast cancer patients reducing fatigue and improving QoL and sleep quality and quantity (Schutz et al., 2021). Moreover, clear evidence in breast cancer survivors indicates a positive effect of exercise on CRF (Matsuoka et al., 2021; Andrioti et al., 2023). However, the dose-response potential of exercise prescription in breast cancer patients remains unclear (Bellissimo Moriah et al., 2023).

Training load monitoring is a concept for enhancing performance and reducing injury risk in athletes (Gabbett and Whiteley, 2017; Gabbett, 2020). Recently, it has been highlighted the importance of transferring the knowledge of training response from athletes to enhance exercise-induced health benefits in patients (Bouça-Machado et al., 2020; Carter et al., 2021; McCarthy et al., 2022). For example, training load monitoring has already been applied to athletes to understand physical fatigue (Halson, 2014). One of the most commonly used training load approaches is through the monitoring of session rating of perceived exertion (sRPE), which is the product of the average intensity (internal load) multiplied by the total duration of the training session (external load) (Foster et al., 2001). The practical application of this approach has been confirmed in various athletic populations, from children to adults, irrespective of expertise level or sports characteristics (Haddad et al., 2017). A simple ratio of acute to chronic training load, called acute-to-chronic workload ratio (ACWR), has been broadly used to guide sports medicine professionals in training planning modifications (Hulin et al., 2016). This ratio quantifies an athlete’s recent training load to a longer-term training load. The ratio itself is defined by dividing the acute by the chronic workload. The most common intervals used in the literature are 7 days for acute and 28 days for chronic workload (Blanch and Gabbett, 2016; Hulin et al., 2016; Carey et al., 2017; Griffin et al., 2020). For performance enhancement and athlete’s health, ACWR is recommended to be between 0.8 and 1.3, reflecting a balance in training load between the last week and the previous month (Gabbett, 2016). Lately, individualization has been promoted in exercise oncology, revealing the potential role of training load monitoring in cancer patients (Carter et al., 2021).

Beyond theoretical and purportedly tailor-made exercise programs, specific protocols with actual effects emerge as evidence supporting the fundamental importance of individualization through ongoing monitoring and adaptations in cancer patients (Carter et al., 2021; Allan et al., 2022). Although there are no specific guidelines for exercise in the cancer patient undergoing active treatment due to the varied and heterogenous nature of cancer types and treatments, adherence to general exercise recommendations is promoted by internationals organizations (Campbell et al., 2019; Hayes et al., 2019; Patel et al., 2019). Thus, despite progress in exercise oncology research for breast cancer patients, training load monitoring is underutilized (Carter et al., 2021; Petrigna et al., 2023). The primary purpose of this research was to retrospectively examine the relationship between consistent training load and physical function and health outcomes among breast cancer patients currently undergoing active anticancer treatment. The study hypothesized that patients who successfully sustained ACWR within a range between 0.8 and 1.2 from week 6 to week 12 would experience improved health outcomes, independent of adherence rate in exercise program.

## 2 Materials and methods

### 2.1 Study design and participants

A pretest-posttest design study was conducted. Enrollment was completed in 1 year, between April 2022 to April 2023. A sample of 19 females with breast cancer was recruited to participate in an exercise program. The study was approved by a university hospital’s scientific and ethics committee (approval number ΕΒΔ89/04–02–2022). The inclusion criteria for study participation required participants to have primary breast cancer (pre- or post-menopausal) and be under active anticancer treatment, including chemotherapy, radiotherapy, immunotherapy, or peri-operative care. Since participants self-reported to the program, medical permission from their oncologist and cardiac clearance from a cardiologist were required. All participants read and signed an informed consent form before enrolling in the program. Patient history and a detailed explanation of the experimental procedures were taken before the exercise program commenced. Other prerequisites for enrollment included a medical report from the oncologist (detailing past and current treatments, other drugs, and histological results) and a complete blood count no older than 3 months before enrollment. All outcome measures were assessed at baseline (T0), at week 6 (T1), and at week 12 (T2) in the same sequence each time.

### 2.2 Intervention

All patients participated in a 12-week training program. The training program was performed at the Attikon University Hospital’s exercise lab. Exercise sessions consisted of 30–45 min of moderate-intensity endurance and resistance training, three times a week. Before and after the main exercise sessions, a 5-min warm-up and a 5-min cool-down procedure were applied. Treadmill (Pro, Woodway, United States), stationary bike (BikeErg, Concept2 Inc., United States), and rowing ergometer (RowErg, Concept2 Inc. United States) were used for aerobic and free weights (dumbbells), resistance bands, and bodyweight exercises were used for strength training. The exercise program was designed and performed by physiotherapists and sports scientists. All sessions were supervised and conducted on a one-on-one basis. The rate of adherence was measured by the ratio of the completed to the planned session’s duration. Before each session, blood pressure with an automatic monitor (BP A1 Easy, Microlife AG, Switzerland) and oxygen saturation (SpO_2_) with a pulse oximeter (CMS50M, Contec Co. Ltd., China) were evaluated. Blood pressure cuffs were set on the unaffected limb. Cut-off values for initiating a session were set at 180 mmHg for systolic blood pressure (SBP), 100 mmHg for diastolic blood pressure (DBP), and 94% SpO_2_. Heart rate (HR) monitoring with a chest strap (H10, Polar Electro, Finland) through the Polar Team app installed in an iPad device and RPE were constantly evaluated during each session. Moderate-intensity aerobic exercise was based on the American College of Sports Medicine recommendations of 64%–76% of age-predicted maximal HR (220–age) or an RPE of 12–13 out of 20 for fatigue (American College of Sports Medicine, 2021). Resistance training intensity was determined based on the RPE, with an aim of 12–13. After each session, session RPE (average RPE multiplied by the duration of the session) was calculated for the training load.

### 2.3 Measurements

#### 2.3.1 Body composition

Body composition was assessed through bioelectrical impedance analysis (BIA; MC-780MA, Tanita Co., Japan). BIA is a commonly used noninvasive method for estimating body composition by measuring the resistance and reactance of electrical flow through the body’s tissues, which varies based on the conductivity of different tissue types such as fat, muscle, and water (Ward, 2019). Before measurement, patients were instructed to be well-hydrated and to fast, abstaining from consuming food and drinks, including coffee, for at least 2–3 h. The bioelectrical impedance analysis measurement extracted body fat percentage (%), fat mass (kg), free-fat mass (kg), muscle mass (kg), and total body water (%). Moreover, phase angle (deg.) and sarcopenic index (kg/m^2^) were analyzed using the device. Body mass index (BMI) was calculated through body weight and standing height.

#### 2.3.2 Physical function

The 6-min walk test (6MWT) and 30-s sit-to-stand (30STS) were performed for physical function determination. 6MWT is a valid and reproducible test in cancer patients (Schmidt et al., 2013). Similarly, 30STS has been revealed as a good indicator of physical fitness in females with breast cancer (Díaz-Balboa et al., 2022). The 6MWT was performed in a 25-m corridor. Patients were instructed to walk at maximum speed for 6 minutes, and during the last 2 minutes, they were asked to maintain their highest speed at a stable rate to cover the greatest possible distance. The total distance covered in meters was recorded. For 30STS, the patient was instructed to complete as many repetitions as possible within 30 s, with only those repetitions being counted in which the thighs or glutes fully touched the chair, and the patient returned to an upright position by extending the knees and hips. The evaluator conducted a demonstration before the evaluation. The total number of completed repetitions was recorded. RPE was assessed for fatigue and dyspnea for both tests at the end of the test. Also, HR monitoring with a chest strap (H10, Polar Electro, Finland) was applied throughout the whole procedure. SpO_2_, SBP, and DBP were measured immediately at the end of each test. From the 6MWT score, the energy cost of walking was estimated from the Physiological Cost Index/Energy Expenditure Index (PCI/EEI). The PCI/EEI is a measure used to quantify the energy required to walk by calculating the ratio of the HR reserved during 6MWT to distance covered [PCI/EEI (beats/meter) = (HR_6MWT_–HR_rest_)/(6MWT meters/6)]. HR rest was measured in a sitting position via a pulse oximeter.

#### 2.3.3 Muscle strength

Isometric muscle assessment was conducted using a hand-held dynamometer (HHD; Muscle Controller, Kinvent, Montpellier, France). We assessed the shoulder abduction, elbow flexion, and extension in a sitting position; chest squeeze in an upright position; knee extension and flexion in a prone lying position; and hip abduction in a supine position. Each isometric exercise was carried out for 6 s, in two sets for each muscle group. The isometric chest squeeze was performed by interpalmar pressing the HHD at chest level, with the elbows out to the sides and the forearms parallel to the floor. All measurements were conducted by the same evaluator to ensure higher reliability (Jackson et al., 2017). Isometric handgrip strength (HG) was performed using a hydraulic grip dynamometer (Lafayette, Model J00105, Lafayette Instrument Co., IN, United States). Participants were instructed to stand upright, with the shoulder at 0° flexion beside their body, the elbow at 90° flexion, and the forearm in a mid-position. They were asked to squeeze the dynamometer to its maximum for about 5 s. Where applicable, both body sides were tested. Statistical analyses were conducted on the affected and unaffected upper limbs based on the side of breast surgery and the dominant and non-dominant lower limbs based on self-reported preference.

#### 2.3.4 Self-reported questionnaires

##### 2.3.4.1 Quality of life

Quality of life was evaluated using the European Organization for the Research and Treatment of Cancer Quality of Life Questionnaire (EORTC QLQ-C30). EORTC QLQ-C30 is a broadly used patient-reported outcome measure (PROM) in women with breast cancer (Gebert et al., 2022). The EORTC QLQ-C30 has been validated in many languages, including Greek (Kontodimopoulos et al., 2011). The questionnaire consists of 30 questions and incorporates five functional scales (physical, role, emotional, social, and cognitive), three symptom scales (fatigue, nausea and vomiting, pain), a global health status/quality of life scale, and several single items assessing additional symptoms commonly reported by cancer patients, such as dyspnea, loss of appetite, insomnia, constipation, diarrhea, and financial difficulties. All scales and single-item measures range in scores from 0 to 100. For the functional scales and the global health status/quality of life scale, a higher score represents a better level of functioning or wellbeing. A higher score for the symptom scales and items indicates more serious difficulties or problems (Aaronson et al., 1993).

##### 2.3.4.2 Sleep quality

Sleep quality was assessed by Pittsburgh Sleep Quality Index (PSQI). PSQI has been translated and validated in many languages, including Greek, especially for cancer patients (Kotronoulas et al., 2011). The PSQI consists of 19 questions, grouped into seven clinically-derived “component” scores. These components are subjective sleep quality, latency, duration, habitual sleep efficiency, sleep disturbances, use of sleeping medication, and daytime dysfunction. Each of these seven component scores is rated on a scale from 0 (no difficulty) to 3 (severe difficulty), and the scores are then summed to provide a global PSQI score, ranging from 0 to 21 (Buysse et al., 1989).

##### 2.3.4.3 Physical activity level

The short form of the International Physical Activity Questionnaire (IPAQ) was used to determine physical activity level. IPAQ is designed to provide comparable data across various settings and has been implemented in many countries, including Greece (Papathanasiou et al., 2009). IPAQ is composed of 7 questions and provides a brief assessment of an individual’s physical activity over the previous 7 days. It categorizes activities into three levels: walking, moderate-intensity, and vigorous-intensity. It considers these activities’ frequency (days per week) and duration (minutes per day). Responses from the IPAQ were used to compute Metabolic Equivalent Task minutes per week (MET-min/week) (Sjostrom et al., 2005).

#### 2.3.5 Heart rate variability

Ultra-short heart rate variability (HRV) was assessed with a six-channel electrocardiogram (ECG; Cardioscan, GmbH, Germany). HRV is dependent on the mean RR interval (or mean HR). A resting ECG was conducted in a standard seated position for 3 minutes, ensuring a controlled and quiet environment (Young and Leicht, 2011). All participants were advised to avoid alcoholic and caffeinated drinks on the morning before the assessment. The root mean square of successive differences (RMSSD) between heartbeats was used as a time-domain HRV index, representing parasympathetic activity. No other parameter was analyzed, as the rest of the HRV indices, including time-domain and frequency-domain parameters, require longer recording (Nussinovitch et al., 2011).

### 2.4 Training load monitoring

Training load monitoring was recorded through a commercially available athlete monitoring system (AMS; Athlete Monitoring, Fitstats Inc., Canada). From the first session, patients were educated about the 10-point RPE scale and how to interpret the average intensity of each session (average RPE). Then, the average RPE (internal load) was multiplied by session duration in minutes (external load) to provide arbitrary units (a.u.) of session training load. A coupled 7:28 rolling average ACWR was calculated automatically by the AMS. An optimal ACWR for health and performance enhancement is between 0.8 and 1.3, based on Hulin et al. (2016). Training session features (RPE and duration) were input into AMS by the exercise provider. Training sessions were designed independently to the chronic training load, based on HR and self-reported RPE during exercise, as previously described. Retrospectively, the sample was divided into two categorical groups based on either maintaining the optimal ACWR (defined as between 0.8 and 1.3; ACWR_0.8-1.3_) between weeks 6 and 12 or falling out of this range (ACWR_out_). The adherence rate was calculated based on exercise sessions reported in AMS concerning session completion and targeted duration.

### 2.5 Statistical analysis

Medians and interquartile ranges (25%–75%; IQR_25-75_) are presented for descriptive statistics. Frequencies and percentages are used for nominal variables. Changes in all dependent variables over time were compared using Friedman’s One-Way Repeated Measure Analysis of Variance by Ranks. Post hoc analyses using the Dunn-Bonferroni test were performed to identify times when changes occurred. The total sample was divided into two groups; those who maintained ACWR within a range of 0.8–1.3 and those who were out of this range from week 6 to week 12. The Mann-Whitney *U* test was used to compare the magnitude of the two groups’ changes after calculating the median of differences (MD) between baseline (T0) to the end of intervention (T2) and week 6 (T1) to the end of intervention (T2). The Wilcoxon signed-rank test was also used for within-group changes between T1 and T2. Effect sizes (ES) and 95% confidence interval for medians (95%CI) were calculated for all parameters. For Friedman’s One-Way Repeated Measure Analysis of Variance by Ranks, Kendall’s W value (Coefficient of concordance; w) was used as effect size, with formula W = *x* ^2^/N(K-1); where W is the Kendall’s W value; x^2^ is the Friedman test statistic value; N is the sample size. For the Mann-Whitney *U* test and Wilcoxon signed-rank test, the effect size r was calculated as Z statistic divided by the square root of the sample size (N) [Z/sqrt(N)]. The interpretation values for ES w and r are set as follows: 0.10 - < 0.3 (small effect), 0.3 - < 0.5 (moderate effect), and ≥0.5 (large effect). Changes between different time points (T0-T1, T1-T2, and T0-T2) for the total sample were also used for Spearman’s rho (*ρ*) correlation coefficient analyses. Statistical significance was set at 5%. Statistical analyses were performed using IBM SPSS software, version 21.0 (Armonk, NY: IBM Corp).

## 3 Results

### 3.1 Demographics

Of the 19 breast cancer patients initially included, one was unable to participate because the medical doctor did not provide permission until active treatment completion, five were fully assessed at T0 but could not follow the program regularly, two were fully evaluated at T0 and T1 but had low adherence rates due to treatment-related adverse events, causing them to stop the program, and one completed the program but did not undergo the final assessment at T2 for personal reasons. Ultimately, a final sample of ten females (49.08 [IQR_25-75_: 44.36–53.65] year, 23.4 [IQR_25-75_: 21.8–29.48] BMI) completed the program and was analyzed. At admission, most patients were at premenopausal status, grade III, HER2 negative, and under radiotherapy/chemotherapy (Table 1). All participants had more than an 80% adherence rate for the whole program, with no statistically significant difference between the two groups. Five women were within the predefined ACWR range (0.8–1.3).

**TABLE 1:** Demographics and baseline body composition. Data are presented as median [interquartile ranges (IQR_25-75_)] or as frequency (percentage). Rounding has been applied in decimals when five or more rounded up, and less than 5 rounded down.

	Total sample (N = 10)	ACWR_0.8-13_ (n = 5)	ACWR_out_ (n = 5)
Age, yrs	49.08 [44.36, 53.65]	51.24 [46.89, 58.79]	44.62 [39.27, 53.1]
Height, cm	165.20 [161.00, 167.00]	165.40 [159.80, 165.75]	165.00 [160.00, 174.10]
Grade, n (%)			
I	2 (20%)	-	2 (40%)
II	5 (50%)	4 (80%)	1 (20%)
III	3 (30%)	1 (20%)	2 (40%)
Menopausal status, n (%)			
Premonopausal	8 (80%)	4 (80%)	4 (80%)
Postmenopausal	2 (20%)	1 (20%)	1 (20)
Tumor receptors, n (%)			
PR (+)	7 (70%)	3 (60%)	4 (80%)
ER (+)	7 (70%)	3 (60%)	4 (80%)
Ki-67 (+)*	4 (40%)	1 (20%)	3 (60%)
HER2 (+)**	3 (30%)	2 (40%)	1 (20%)
Current treatment, n (%)			
Chemo/Radiotherapy	9 (90%)	4 (80%)	5 (100%)
Immunotherapy	1 (10%)	1 (20%)	-
IPAQ, MET-minute/week	1041.75 [810.38, 2235.75]	1971.00 [1217.75, 3057.00]	967.50 [318.0, 1041.75]
Body composition			
BMI, kg/m^2^	23.40 [21.80, 29.48]	23.30 [21.30, 26.55]	23.50 [20.60, 30.30]
Fat, %	33.75 [24.70, 40.88]	33.80 [22.45, 39.00]	33.70 [23.40, 41.35]
FFM, kg	44.50 [41.22, 48.53]	42.20 [41.05, 45.25]	48.50 [41.55, 48.60]
TBW, %	47.25 [42.15, 53.30]	47.00 [43.30, 55.05]	47.50 [41.80, 54.50]
Phase angle, degrees	4.80 [4.48, 5.30]	4.90 [4.60, 5.25]	4.50 [4.40, 5.50]
Sarcopenic index, kg/m^2^	6.54 [6.06, 6.87]	6.39 [6.01, 6.77]	6.68 [6.11, 7.19]

ACWR_0.8-13_ = those who maintained ACWR within a range of 0.8–1.3 between weeks 6 and 12; ACWR_out_ = those who did not maintain ACWR within the predefined range between weeks 6 and 12; BMI = Body Mass Index; FFM = Free-Fat Mass; TBW = Total Body Water; PR = Progesterone-receptors; ER = estrogen-receptors; HER2 = human epidermal growth factor receptor 2; Ki-67* = non-available data for three women; HER2 (+)** = non-available data for one woman.

### 3.2 Within-subject analysis

Repeated-measures Friedman test for within-subject analysis revealed statistically significant changes for body weight (*p* = 0.041), 6MWT (*p* = 0.006), 30STS (*p* = 0.001), elbow flexion of the affected (*p* = 0.032), and shoulder abduction for the unaffected limb (*p* = 0.032) (Table 2). Post-hoc Dunn-Bonferroni analysis demonstrated that all changes occurred between T0 and T2 except for shoulder abduction. Large effect size was observed for 6MWT (ES_r_ = 0.52) and 30STS (ES_r_ = 0.68), and moderate effect size for body weight (ES_r_ = 0.32), knee extension of the dominant limb (ES_r_ = 0.31, *p* = 0.062), elbow flexion of the affected limb (ES_r_ = 0.38), and shoulder abduction of the unaffected limb (ES_r_ = 0.38).

**TABLE 2:** The median [95% confidence intervals for the median] and effect size for each outcome measure are presented for within-subjects repeated-measure analysis (N = 10).

Outcome measure	T0 [95%CI]	T1 [95%CI]	T2 [95%CI]	ES (w)	*p*-value
Body composition					
Body weight, kg	65.90 [55.60, 78.70]	65.85 [53.20, 76.90]	65.45 [54.50, 75.20]	*0.32*	0.041 *
Fat, %	33.75 [18.10, 42.30]	31.50 [21.60, 40.10]	32.60 [ 17.80, 39.00]	0.20	0.132
FFM, kg	44.50 [40.70, 48.60]	43.35 [41.40, 49.00]	45.05 [41.50, 47.40]	0.13	0.273
BMI, kg/m^2^	23.40 [20.30, 30.00]	23.40 [19.40, 29.70]	23.40 [19.90, 29.10]	0.18	0.172
TBW, %	47.25 [41.10, 58.40]	48.70 [42.60, 55.80]	48.05 [43.40, 58.50]	0.17	0.179
Phase angle, degrees	4.80 [4.40, 5.30]	5.05 [4.50, 5.60]	4.85 [4.50, 5.50]	0.28	0.058
Sarcopenic index, km/m^2^	6.54 [5.94, 7.17]	6.45 [5.91, 7.27]	6.40 [6.06, 7.46]	0.09	0.407
Quality of Life (EORTC-QLQ-C30)					
Functional scales, %	81.67 [68.34, 92.00]	83.50 [65.33, 91.67]	82.33 [67.00, 93.67]	0.008	0.924
Symptoms scales, %	16.67 [7.41, 24.08]	18.52 [9.26, 29.63]	23.15 [7.41, 46.29]	0.10	0.358
Global health status, %	79.17 [50.00, 83.33]	66.67 [50.00, 91.67]	66.67 [33.33, 100.00]	0.09	0.402
Sleep Quality					
PSQI, score	5.00 [5.00, 8.00]	6.00 [4.00, 8.00]	5.00 [4.00, 8.00]	0.003	0.969
Resting vital signs					
HR, b/min	68.5 [59.0, 76.0]	68.0 [63.0, 75.0]	69.0 [58.0, 79.0]	0.13	0.283
SpO_2_, %	98.0 [97.0, 99.0]	98.0 [97.0, 99.0]	98.0 [97.0, 99.0]	0.01	0.867
SBP, mmHg	116.0 [107.0, 126.0]	106.0 [101.0, 112.0]	114.5 [97.0, 123.0]	0.26	0.071
DBP, mmHg	74.0 [70.0, 86.0]	67.5 [63.0, 74.0]	69.0 [60.0, 80.0]	0.26	0.078
HRV					
RMSSD, ms	20.5 [17.0, 47.0]	28.5 [15.0, 41.0]	21.5 [12.0, 46.0]	0.10	0.358
Physical function					
6MWT, m	569.0 [530.0, 622.0]	596.0 [573.0, 673.0]	628.5 [579.0, 715.0]	0.52	0.006 *
6MWT-HR, b/min	140.5 [124.0, 149.0]	141.0 [123.0, 167.0]	147.5 [126.0, 160.0]	0.008	0.926
6MWT-RPE_fatigue_	11.0 [7.0, 15.0]	14.0 [11.0, 15.0]	13.0 [11.0, 15.0]	0.17	0.177
6MWT-RPE_dyspnea_	7.0 [6.0, 12.0]	6.5 [6.0, 13.0]	6.0 [6.0, 10.0]	0.06	0.248
PCI/EEI	0.776 [0.588, 0.872]	0.709 [0.586, 0.887]	0.713 [0.506, 0.825]	0.12	0.301
30STS, reps	12.0 [9.0, 18.0]	12.5 [11.0, 18.0]	13.5 [11.0, 19.0]	0.68	0.001 *
Muscle strength					
Handgrip (affected), kg	26.0 [22.0, 30.0] ^**‡**^	25.0 [23.0, 28.5] ^**‡**^	29.0 [24.0, 31.0] ^**‡**^	0.22	0.11
Handgrip (unaffected), kg	29.0 [26.0, 32.0] ^**‡**^	29.0 [25.0, 33.0] ^**‡**^	32.0 [26.0, 35.0] ^**‡**^	0.28	0.058
Knee flexion (dominant), kg	106.9 [81.0, 155.9]	134.6 [102.0, 149.2]	137.0 [107.0, 177.0] ^**‡**^	0.26	0.097
Knee flexion (non-dominant), kg	118.5 [90.7, 153.0]	122.0 [100.5, 155.0]	129.9 [102.0, 167.0] ^**‡**^	0.04	0.717
Knee extension (dominant), kg	188.0 [133.8, 230.3]	200.0 [147.0, 270.0]	203.0 [150.1, 299.0]	*0.31*	0.062
Knee extension (non-dominant), kg	179.0 [135.3, 243.0]	188.0 [173.0, 235.4]	188.0 [165.7, 262.0]	0.06	0.581
Hip abduction (dominant), kg	146.0 [126.2, 186.9]	152.0 [121.0, 166.5]	144.0 [117.9, 174.0] ^**‡**^	0.01	0.895
Hip abduction (non-dominant), kg	144.0 [121.4, 168.0]	136.6 [132.0, 175.9]	143.0 [107.0, 158.0] ^**‡**^	0.09	0.459
Elbow flexion (affected), kg	157.6 [130.5, 163.6]	162.0 [137.2, 175.5]	174.0 [158.3, 189.0] ^**‡**^	*0.38*	0.032 *
Elbow flexion (unaffected), kg	155.0 [132.1, 172.1]	149.5 [133.5, 177.5]	163.0 [149.7, 168.0] ^**‡**^	0.23	0.121
Elbow extension (affected), kg	114.6 [91.2, 125.1] ^**‡**^	117.0 [97.1, 125.4]	119.0 [97.0, 129.5]	0.12	0.328
Elbow extension (unaffected), kg	121.3 [83.5, 138.0] ^**‡**^	120.0 [105.0, 132.0]	124.0 [101.8, 148.0]	0.04	0.717
Shoulder abduction (affected), kg	123.2 [102.1, 143.6]	128.3 [107.0, 154.0]	139.0 [128.0, 149.1]	0.23	0.121
Shoulder abduction (unaffected), kg	113.8 [91.1, 125.8]	133.0 [104.0, 142.0]	123.3 [103.0, 145.0]	*0.38*	0.032 †
Chest squeeze, kg	139.0 [92.3, 158.4]	143.0 [115.9, 177.8]	146.6 [102.6, 201.1]	0.15	0.264

T0 = baseline; T1 = week 6; T2 = week 12; ES, Kendall’s W effect size; FFM, free-fat mass; BMI, body mass index; TBW, total body water; PSQI, pittsburgh sleep quality index; HR, heart rate; SBP, systolic blood pressure; DBP, diastolic blood pressure; HRV, heart rate variability; 6MWT, 6-min walk test; RPE, rating of perceived effort; PCI/EEI, Physiological Cost Index/Energy Expenditure Index; 30STS, 30-s sit-to-stand; Bold = large effect size; *Italic* = medium effect size; * = Statistically significant difference between baseline to 12 weeks; † = Statistically significant difference between baseline to 6 weeks; ‡ = Statistically significant difference between limbs (muscle strength).

### 3.3 Group comparisons

For the two groups based on ACWR categories, within-group, and between-group comparisons are presented in Table 3. Among all variables, only MET-minute/week (*p* = 0.032) and HR at rest (*p* = 0.016) revealed a statistical significance between the two groups at baseline. Sleep quality improved for ACWR_0.8-1.3_ compared to ACWR_out_ between T0 and T2 (ES_r_ = −0.78, *p* = 0.016). Similarly, large effect size differences among groups presented at T2 (ES_r_ = −0.58, *p* = 0.095) and T1-T2 phase (ES_r_ = −0.50, *p* = 0.151), representing a greater overall improvement between week 6 to week 12, while not statistically significant. EORTC-QLQ-C30 demonstrated a large effect size difference between groups at T2 for functional scales (ES_r_ = 0.76, *p* = 0.016) and global health status (ES_r_ = 0.50, *p* = 0.151), in favor of patients who maintained ACWR within the predefined range. Also, a statistically significant difference in functional scales was observed for each group’s overall change between T0 and T2 (ES_r_ = 0.83, *p* = 0.008). Physical function improved accordingly within each group through 6MWT (ACWR_0.8-1.3_: T0: 554 m [95%CI: 469–675] to T2: 650 m [95%CI: 475–775], ES_r_ = 0.84, *p* = 0.015; ACWR_out_: 582 m [95%CI: 530–622] to T2: 600 m [95%CI: 579–715], ES_r_ = 0.28, *p* = 0.247) and 30STS (ACWR_0.8-1.3_: T0: 12 reps [95%CI: 11–18] to T2: 16 reps [95%CI: 12–20], ES_r_ = 0.859, *p* = 0.014; ACWR_out_: T0: 11 reps [95%CI: 8–18] to T2: 13 reps [95%CI: 10–19], ES_r_ = 0.514, *p* = 0.076), but without reaching statistical significance difference among groups in any phase. Handgrip (ES_r_ = 0.37, *p* = 0.31) and elbow flexion (ES_r_ = 0.31, *p* = 0.413) of the affected limb were improved to a greater extent between T1 and T2 for those who kept their training load steady concerning previous weeks.

**TABLE 3:** The median [95% confidence intervals for the median] and effect size for each outcome measure are presented for within-subjects changes (weeks 6–12) and for the between-subjects magnitude of change (n = 5 for each group).

	ACWR_0.8-1.3_					ACWR_out_					Between-group ES_r_		
Outcome measure	T0 [95%CI]	T1 [95%CI]	T2 [95%CI]	ES_w_	*p*-value	T0 [95%CI]	T1 [95%CI]	T2 [95%CI]	ES_w_	*p*-value	T2	T2–T1	T2–T0
Body composition													
Body weight, kg	61.5 [55.6, 78.7]	60.4 [53.2, 76.9]	59.8 [54.5, 74.5]	*0.41*	0.128	73.3 [47.6, 81.6]	70.9 [47.9, 80.9]	69.8 [47.7, 79.2]	*0.48*	0.091	*−0,36*	*0,43*	0.03
Fat, %	33.8 [18.0, 42.9]	30.3 [21.6, 42.4]	28.9 [17.8, 39.2]	0.16	0.449	33.7 [18.1, 42.3]	34.4 [18.8, 40.1]	33.1 [15.7, 39.0]	0.28	0.241	−0.10	−0.17	−0.03
FFM, kg	42.2 [40.7, 45.6]	42.1 [41.4, 44.3]	42.9 [41.5, 45.3]	0.16	0.49	48.5 [39.0, 48.6]	46.5 [38.9, 49.2]	45.9 [40.2, 49.3]	0.12	0.549	−0,50	0.13	0.03
BMI, kg/m^2^	23.3 [20.3, 29.3]	23.0 [19.4, 28.6]	23.0 [19.9, 27.7]	*0.41*	0.128	23.5 [18.1, 30.6]	24.1 [18.3, 30.8]	23.8 ]18.2, 30.5]	*0.36*	0.165	−0.17	*0,37*	−0.17
TBW, %	47.0 [40.4, 58.5]	49.5 [40.8, 55.8]	50.2 [43.2, 58.5]	0.095	0.623	47.5 [41.1, 58.4]	47.0 [42.6, 57.8]	47.4 [43.4, 60.2]	0.28	0.247	0.10	0.13	0.03
Phase angle, degrees	4.9 [4.5, 5.3]	5.2 [4.5, 5.6]	4.9 [4.8, 5.5]	0.54	0.066	4.5 [4.4, 5.7]	4.5 [4.5, 5.6]	4.7 [4.5, 5.5]	0.13	0.526	*0,30*	−0.17	0.20
Sarcopenic index, km/m^2^	6.39 [5.92, 6.77]	6.24 [5.91, 6.73]	6.32 [6.06, 6.88]	0.12	0.549	6.68 [5.94, 7.20]	6.90 [5.91, 7.55]	6.48 [6.03, 7.63]	0.12	0.549	−0.17	0.03	−0.17
Quality of life (EORTC-QLQ-C30)													
Functional scales, %	80.67 [68.34, 92.00]	86.33 [83.33, 93.33]	84.33 [81.33, 100.00]	0.516	0.076	82.67 [65.33, 95.00]	67.67 [61.00, 91.66]	78.67 [41.33, 83.33]	0.453	0.104	0,76‡	0.17	0,83‡
Symptoms scales, %	9.26 [7.41, 22.22]	16.67 [9.26, 22.22]	35.19 [7.41, 55.56]	*0.032*	0.854	22.22 [7.41, 37.04]	27.78 [16.67, 40.74]	35.19 [7.41, 55.56]	0.221	0.331	*−0,33*	0.03	−0.1
Global health status, %	83.33 [66.67, 91.67]	83.33 [66.67, 100.00]	83.33 [58.33, 100.00]	0.0	1.0	75.00 [33.33, 83.33]	50.00 [33.33, 66.67]	66.67 [33.33, 75.00]	*0.365*	0.161	0,50	−0.27	0.3
Sleep quality													
PSQI, score	6.0 [5.0, 8.0]	6.0 [4.0, 8.0]	5.0 [2.0, 7.0]	*0.424*	0.12	5.0 [4.0, 9.0]	6.0 [5.0, 8.0]	8.0 [5.0, 9.0]	*0.333*	0.189	−0.58	−0.50	−0.78‡
HRV													
RMSSD, ms	18.0 [16.0, 33.0]	19.0 [15.0, 36.0]	19.0 [11.0, 37.0]	0.278	0.249	44.0 [17.0, 75.0]	35.0 [15.0, 183.0]	38.0 [17.0, 82.0]	0.04	0.819	*−0.43*	−0.17	*−0.33*
Physical function													
6MWT, m	554.0 [469.0, 675.0]	604.0 [506.0, 758.0]	650.0 [475.0, 775.0]	0.84	0.015*	582.0 [530.0, 622.0]	580.0 [573.0, 673.0]	600.0 [579.0, 715.0]	0.28	0.247	0.23	0.03	*0.30*
PCI/EEI	0.678 [0.498, 0.910]	0.633 [0.510, 0.887]	0.665 [0.392, 0.821]	0.12	0.549	0.829 [0.742, 0.872]	0.847 [0.662, 1.033]	0.810 [0.705, 0.890]	0.12	0.549	−0.63	*−0.30*	−0.17
30STS, reps	12.0 [11.0, 18.0]	14.0 [11.0, 18.0]	16.0 [12.0, 20.0]	0.859	0.014*	11.0 [8.0, 18.0]	11.0 [11.0, 18.0]	13.0 [10.0, 19.0]	0.514	0.076	*0.37*	*0.31*	0.27
Muscle strength													
Handgrip (affected), kg	25.0 [22.0, 30.0]	24.0 [23.0, 28.5]	30.0 [22.0, 32.0]	0.278	0.249	26.0 [21.0, 30.0]	28.0 [24.0, 31.0]	28.0 [24.0, 31.0]	*0.312*	0.210	−0.03	*0.37*	0.03
Handgrip (unaffected), kg	28.0 [26.0, 33.0]	28.0 [25.0, 30.0]	32.0 [26.0, 32.0]	*0.389*	0.143	30.0 [22.0, 32.0]	31.0 [24.0, 35.0]	33.0 [25.0, 36.0]	0.958	0.008*	−0.50	0.13	−0.27
Knee flexion (dominant), N	121.8 [98.7, 155.9]	105.2 [89.7, 149.2]	121.0 [80.9, 188.0]	*0.36*	0.165	97.4 [73.3, 161.0]	143.85 [134.6, 170.0]	142.65 [110.0, 177.0]	0.188	0.472	−0.15	0.54	0.08
Knee flexion (non-dominant), N	107.0 [68.4, 167.0]	117.6 [65.9, 194.6]	120.0 [57.2, 184.0]	0.12	0.549	122.65 [106.9, 153.0]	138.35 [112.0, 155.0]	137.95 [105.0, 167.0]	0.0	1.0	−0.15	−0.15	0.00
Knee extension (dominant), N	188.0 [139.4, 230.3]	200.0 [194.0, 245.8]	203.0 [145.0, 230.0]	0.28	0.247	175.2 [87.4, 373.3]	208.5 [143.0, 288.3]	225.5 [150.1, 320.0]	*0.438*	0.174	−0.15	*−0.46*	*−0.31*
Knee extension (non-dominant), N	179.0 [118.9, 243.0]	184.0 [162.9, 235.4]	188.0 [125.0, 262.0]	0.2	0.368	185.9 [135.3, 382.6]	209.0 [173.0, 273.6]	216.0 [165.7, 304.0]	0.0	1.0	0.23	−0.12	0.08
Hip abduction (dominant), N	146.0 [131.0, 186.9]	156.0 [144.0, 166.5]	160.0 [138.1, 178.0]	0.16	0.449	142.55 [119.0, 189.5]	133.45 [104.0, 191.0]	130.95 [116.0, 168.8]	0.562	0.105	*0.39*	0.15	0.70‡
Hip abduction (non-dominant), N	144.0 [121.4, 168.0]	135.0 [132.0, 175.9]	143.0 [99.0, 158.0]	0.04	0.819	143.2 [103.8, 208.4]	138.8 [118.0, 202.0]	132.5 [107.0, 166.9]	0.25	0.368	0.00	0.15	0.23
Elbow flexion (affected), N	155.0 [106.0, 172.1]	142.0 [124.2, 177.5]	165.0 [119.0, 184.0]	*0.36*	0.165	151.4 [132.4.176.8]	159.75 [137.0, 182.0]	156.5 [149.7, 166.9]	0.25	0.368	0.23	*0.31*	0.15
Elbow flexion (unaffected), N	160.0 [130.5, 163.6]	169.6 [137.2, 175.5]	174.0 [162.0, 189.0]	0.52	0.074	154.35 [120.7, 221.0]	156.35 [137.0, 218.0]	167.65 [147.0, 190.5]	0.25	0.368	0.15	0.15	*0.31*
Elbow extension (affected), N	123.0 [75.1, 143.6]	121.3 [82.5, 142.9]	111.0 [100.0, 171.0]	0.12	0.549	112.15 [101.0, 123.3]	117.5 [105.0, 130.0]	133.4 [101.0, 148.0]	0.188	0.472	−0.23	−0.15	0.00
Elbow extension (unaffected), N	114.6 [75.7, 131.0]	117.0 [95.4, 125.4]	116.0 [97.0, 179.0]	0.095	0.623	104.1 [91.2, 125.1]	117.5 [97.1, 132.0]	122.0 [91.1, 129.5]	0.188	0.472	−0.16	0.08	0.08
Shoulder abduction (affected), N	125.0 [91.1, 138.0]	115.5 [104.0, 152.8]	125.0 [103.0, 168.0]	0.12	0.549	110.85 [79.6, 116.0]	138.85 [102.0, 142.0]	122.5 [100.4, 129.4]	1.0	0.018†	*0.39*	*0.38*	−0.15
Shoulder abduction (unaffected), N	128.0 [101.3, 153.0]	126.0 [107.0, 157.4]	141.0 [134.0, 157.0]	*0.36*	0.165	118.35 [102.1, 134.1]	129.15 [101.0, 154.0]	128.5 [70.8, 149.1]	0.25	0.368	*0.46*	*0.31*	0.08
Chest squeeze, N	126.0 [92.3, 155.7]	143.0 [133.6, 177.8]	146.6 [111.7, 201.1]	*0.48*	0.091	155.2 [79.6, 164.8]	131.9 [89.6, 178.0]	141.9 [53.5, 201.4]	0.0	1.0	0.08	0.08	0.23

T0 = baseline; T1 = week 6; T2 = week 12; ES_w_, Kendall’s W effect size; ES_r_, Mann-Whitney *U* test effect size (positive values: ACWR_0.8-1.3_ > ACWR_out_); FFM, free-fat mass; BMI, body mass index; TBW, total body water; PSQI, pittsburgh sleep quality index; HR, heart rate; SBP, systolic blood pressure; DBP, diastolic blood pressure; HRV, heart rate variability; 6MWT, 6-min walk test; RPE, rating of perceived effort; PCI/EEI, Physiological Cost Index/Energy Expenditure Index; 30STS, 30-s sit-to-stand; Bold = large effect size; *Italic* = medium effect size; * = Statistically significant difference between baseline to 12 weeks; † = Statistically significant difference between baseline to 6 weeks; ‡ = Statistically significant difference in between-groups comparisons.

### 3.4 Correlations

A positive correlation between age and grade diagnosis was detected (*ρ* = 0.748, *p* = 0.02). FFM increases between T1 and T2 positively correlated with global health status (*ρ* = 0.692, *p* = 0.039). PSQI score negative correlation with handgrip strength of the affected limb (*ρ* = -0.769, *p* = 0.015) and ACWR (*ρ* = -0.667, *p* = 0.05) was demonstrated, indicating that a greater change of handgrip strength and a higher ACWR between T1 and T2 is associated with better sleep quality. 6MWT was not associated with ACWR in any phase, but a relationship with global health status change between T0 to T2 among both variables was demonstrated (*ρ* = 0.689, *p* = 0.04). Additionally, a positive strong correlation between changes from T1 to T2 for knee extensors of the dominant limb and the PCI/EEI (*ρ* = 0.933, *p* < 0.001), but not for the non-dominant leg (*ρ* = 0.586, *p* = 0.097) was revealed. No correlation was observed between knee extensors and sit-to-stand changes at any time. Elbow flexors and handgrip strength changes between T1 and T2 were also associated (*ρ* = 0.658, *p* = 0.054 unaffected elbow-unaffected handgrip; *ρ* = 0.785, *p* = 0.012 affected elbow-unaffected handgrip; *ρ* = 0.814, *p* = 0.008 unaffected elbow-affected handgrip; *ρ* = 0.797, *p* = 0.01 affected elbow-affected handgrip). No other significant plausible correlation between variables was found.

## 4 Discussion

The primary objective of this study was to retrospectively assess the association between maintaining a balanced training load and physical and health outcomes in breast cancer patients undergoing active therapy. The findings suggest that patients who can maintain a stable ACWR from week 6 to week 12, compared to previous weeks, may experience improved physical and health outcomes, regardless of their adherence to the exercise program. A 12-week exercise program appears effective in preserving or enhancing physical function, although its impact on other health outcomes remains unclear. However, sleep quality, QoL, and muscle strength of the operated limb are perhaps affected significantly by the ACWR in breast cancer undergoing treatment, indicating a trend of more significant improvements for those who manage to maintain an ACWR within the range of 0.8–1.3 from week 6 onwards. Overall, while adherence to an exercise program during active treatment seems to help maintain breast cancer patients’ physical and health status, a more sophisticated approach to training load monitoring could potentially augment these improvements.

Recently, researchers proposed that the transfer of knowledge from elite athletes and adaptation of training principles to chronic disease patients may have a meaningful impact on individualization and enhancing clinical outcomes (Carter et al., 2021; McCarthy et al., 2022). A viewpoint article by Carter et al. (2021) highlighted the role of training load monitoring and management through wearable devices. They recommend regular monitoring of internal and external loads to evaluate a cancer patient’s psychopathological adaptation to exercise training (Carter et al., 2021). Although physical activity level through wearable devices is the most commonly evaluated outcome measure in oncological practice (Beauchamp et al., 2020), no study of training load monitoring in cancer patients has ever been conducted, according to our knowledge. Our findings from this pilot study enhance the concept of training load monitoring via AMS and a new field is opening for future research on breast cancer patients undergoing active treatment.

Endurance training has a significant impact on cardiovascular fitness and stamina. It improves overall stamina and the ability of the heart to pump oxygenated blood, both in individuals with and without prior cardiovascular disease (Meka et al., 2008). Specifically, endurance training increases VO_2_max, enhances maximal cardiac output, and improves ventricular function (Hagberg et al., 1989; Hellsten and Nyberg, 2015). For instance, endurance exercise training leads to positive adaptations in the heart, such as increased end-diastolic dimension and improved resistance to ischemia (Moore et al., 1998). It also improves cardiac and skeletal muscle oxidative metabolism, particularly beneficial for heart failure patients (Ventura-Clapier et al., 2007). Additionally, endurance training increases skeletal muscle myoglobin concentration and fatty acid oxidation, further enhancing cardiovascular fitness (Braun et al., 1991). Moreover, endurance training can lead to a cardioprotective phenotype, increasing the heart’s resistance to injury during ischemia-reperfusion events and enhancing the expression of superoxide dismutase in cardiac mitochondria (Powers et al., 2014). Similarly, high-intensity interval training, a form of endurance training, has also been shown to have a large beneficial effect on VO_2_max (Milanović et al., 2015). In contrast, resistance training focuses on muscular strength, and while it has favorable effects on cardiovascular function and metabolism, it can reduce central arterial compliance in healthy men, which is contrary to the beneficial effect of regular aerobic exercise (Miyachi et al., 2004). However, resistance training combined with impact loading have been revealed as the most beneficial mode of exercise for prostate cancer receiving androgen deprivation therapy (Newton et al., 2019). These findings underscore the diverse benefits of different types of training on cardiovascular health and physical fitness, highlighting the importance of selecting the appropriate training regimen based on individual health goals and conditions.

It is well-established that participating regularly in exercise programs during anticancer treatment has a great impact from the molecular regulation to patient-reported outcome measures (PROM). Although several potential molecular and cellular pathways have been proposed in the literature for cancer patients, such as muscle-derived factors (myokines) (Spiliopoulou et al., 2021; Papadopetraki et al., 2022), less telomere attrition (Nomikos et al., 2018), and obesity-related hormones (Adraskela et al., 2017), the clinical and patient-reported outcome measures (PROM) might matter most for the patients. Some of the most used PROM in cancer research are cancer-related fatigue, sleep quality, and self-reported quality of life. Sleep quality and fatigue in breast cancer patients undergoing chemotherapy are closely related, indicating a common underlying mechanism (Liu et al., 2012; Imanian et al., 2019). However, no correlation between the PSQI score and EORTC-QLQ-C30 domain changes was observed in our study.

Sleep disorders are highly prevalent among breast cancer patients undergoing chemotherapy or radiotherapy (Fontes et al., 2017; Imanian et al., 2019; Grayson et al., 2022). Almost 40% of breast cancer patients in a radiotherapy department suffer from sleep disturbances (Wang et al., 2021). Most studies evaluating subjective sleep quality use PSQI, which has proven internal consistency reliability and construct validity for cancer patients (Beck et al., 2004). Previous studies have found improvements in subjective sleep quality measured via PSQI for those who participated in a physical activity intervention (Rogers et al., 2017; Kreutz et al., 2019). However, it remains uncertain if exercise training has any real effect on subjective and objective sleep parameters (Mercier et al., 2017). This uncertainty can be attributed to the chronobiology of exercise. For example, while our training program was delivered mainly in the morning, existing evidence suggests a greater cardiac effect of evening training programs, which last during sleeping (Brito et al., 2022). Furthermore, prior research has shown no exercise effect on post-anticancer treatment (Lahart et al., 2018), which questions the value of intervening after the completion of active treatment. Our results suggest improving sleep quality only for those who maintained ACWR within predefined ranges throughout the study. Therefore, it might be vital to intervene during active treatment to improve sleep quality, using training load monitoring and delivering tailor-made exercise training programs.

Physical function is a thoroughly researched aspect of exercise oncology. The 6MWT has been proposed as a measure of health in breast cancer patients due to its close association with health-related QoL (Galiano-Castillo et al., 2016). A recent systematic review with meta-analysis showed that the total distance walked was significantly reduced in breast cancer survivors compared to healthy controls (But-Hadzic et al., 2021). The minimal clinically important difference (MCID) for the 6MWT conducted on a treadmill among physically active women diagnosed with breast cancer was estimated to be roughly 54 m during chemotherapy and 41.6 m after treatment (Cantarero-Villanueva et al., 2023). However, in most exercise studies, 6MWT improvements were observed during the active treatment when patients participated in a concurrent training program (Correia et al., 2023; Trommer et al., 2023). Similarly, the 30STS has been found to correlate closely with 6MWT and serves as a helpful tool for estimating cardiorespiratory fitness (VO2max) in breast cancer patients (Díaz-Balboa et al., 2022). It also effectively stratifies survivors based on cancer-related fatigue (Cuesta-Vargas et al., 2019). In the POWER program, which involved cancer patients undergoing active treatment, there was an improvement of nearly two repetitions in the 30STS after the exercise intervention (Coletta et al., 2021). Consistent with previous research, our study demonstrates a significant effect size improvement for 6MWT and an increase of about two repetitions for 30STS in the 12-week training program. When considering ACWR, the enhancement in physical function was even more pronounced for those who achieved a balanced ACWR from week 6 onwards. Hence, participating in an exercise program during active treatment not only prevents the decline of physical function but also leads to notable clinical improvements.

The therapeutic modalities used for breast cancer management, including surgery, chemotherapy, radiation, targeted therapy, and hormone therapy, have been associated with multiple consequences, including an adverse impact on muscle function. While decreases in physical function reduce general activity, muscle strength loss reduces functionality, which necessitates more external assistance and reduces the patient’s independence, resulting in a decline in health-related QoL (Kärki et al., 2005). Thus, assessing and enhancing muscle strength is crucial in clinical practice (Campbell et al., 2012). Indeed, due to this treatment-induced muscle dysfunction, exercise training has been recommended to initiate as soon as possible after cancer diagnosis (Klassen et al., 2017). We observed a trend towards significant improvement in handgrip and elbow flexor strength of the affected limb after 6 weeks of regular training, and these improvements correlated with each other. Despite our thorough investigation into upper and lower limb isometric muscle strength, no other significant changes were detected.

Physical activity levels and exercise program participation can maintain or improve QoL in breast cancer patients undergoing active treatment and survivors. For example, breast cancer females under chemotherapy demonstrate a positive correlation between the physical functioning of EORTC-QLQ-C30 and total energy expenditure measured by IPAQ (Maridaki et al., 2020). Breast cancer patients also have a lower level of moderate-to-vigorous physical activity (Irwin et al., 2004; Gal et al., 2019; Maridaki et al., 2020). Our sample was considered sedentary based on baseline MET-minutes/week (IPAQ = 1041.75 [IQR_25-75_ 810.38, 2235.75]). Exercise training also can potentially affect QoL in a manner specific to the cancer site, though this connection remains uncertain (Furmaniak et al., 2016). In a recent study on Greek breast cancer survivors, QoL measured through EORTC-QLQ-C30 improved after an 8-week home-based tele-exercise program (Andrioti et al., 2023). However, active treatment leads to deterioration of QoL aspects (Zamel et al., 2021), so just maintaining a steady state in these patients and preventing unfavorable treatment effects might also be considered beneficial. EORTC-QLQ-C30 was not improved in any aspect across time points for the total sample. However, a statistically significant increase in function and global health was revealed for those who maintained a stable ACWR from the mid-point to the end of the intervention.

Training load monitoring through ACWR has many practical implications in clinical practice. For example, a breast cancer patient under chemotherapy might report a 7 out of 10 relative RPE for a 20-min session. The same patient, a month before starting active treatment, could assess a 40-min session of 4 RPE scale. While in arbitrary units there is a 12.5% difference between these sessions (140 vs. 160 arbitrary units), in clinical practice and in terms of ACWR, these relative sizes are almost identical, maintaining balance in training load. Thus, using a model which includes internal and external ratios, such as ACWR, can counterbalance the self-reported unfavorable effects during active treatment by comparing the relative loads with the patient’s physical and health status at the cancer care continuum, from healthy tο exhausted phases.

### 4.1 Limitations

Every research study has limitations. Firstly, our study is a pilot study with a small sample size of a newly delivered approach in the cancer population, providing promising findings for future research. Also, it is a single-group study with a non-homogenous sample making the direct interpretation of our intervention difficult. Conducting research in oncology is challenging, especially in countries without prior studies on the topic. To our knowledge, our investigation is the first interventional study on exercise training in breast cancer patients undergoing active treatment in Greece. Third, prediction equations of bioelectrical impedance analysis devices have shown meaningful differences in body composition compared to dual-energy x-ray absorptiometry (DXA) (Bell et al., 2020; Baş et al., 2023). Fourth, ATS Committee on Proficiency Standards for Clinical Pulmonary Function Laboratories (2002) recommends a 30-m corridor to conduct 6MWT. However, we could only use a 25-m corridor, which can lead to an underestimation of the total covered distance. Moreover, we used an indirect estimation of physical activity level via a subjective self-reported questionnaire (IPAQ), threatening a recall bias and an overestimation of self-reported levels of physical activity, as has been reported in cancer patients undergoing chemotherapy (Vassbakk-Brovold et al., 2016). Following this study limitation, it is impossible to interpret our intervention concerning individual daily/weekly physical activity. Also, an ultra-short HRV might be too short to detect changes accurately, especially for time-domain indices. Lastly, we used a 7:28 rolling average ACWR, while shorter periods of acute and chronic intervals might be more useful in cancer patients undergoing treatment due to the regular treatment-induced health fluctuations.

In conclusion, this study underscores the significance of monitoring and maintaining a balanced training load, specifically a stable ACWR, in breast cancer patients undergoing active treatment. Our findings indicate that the ability to maintain an ACWR within the range of 0.8–1.3 from week 6 onwards may lead to superior physical and health outcomes, such as 6MWT, 30STS, and sleep quality. Notably, this is the case even when accounting for adherence to the exercise program, which plays a crucial role in preserving or enhancing physical functionality. Moreover, other critical parameters, such as QoL and muscle strength of the operated limb, seem to be particularly influenced by the ACWR. While the importance of consistent exercise during active treatment is evident, the data suggest that integrating a refined approach to training load monitoring can further enhance the physical and health outcomes for breast cancer patients. It is paramount for clinicians to consider both program adherence and precise load management to optimize therapy outcomes for breast cancer patients undergoing active treatment.

## Data Availability

The raw data supporting the conclusion of this article will be made available by the authors, without undue reservation.
